# Leukocyte expression profiles reveal gene sets with prognostic value for seizure-free outcome following stereotactic laser amygdalohippocampotomy

**DOI:** 10.1038/s41598-018-37763-5

**Published:** 2019-01-31

**Authors:** Ryan Sprissler, Robert Bina, Willard Kasoff, Marlys H. Witte, Michael Bernas, Christina Walter, David M. Labiner, Branden Lau, Michael F. Hammer, Martin E. Weinand

**Affiliations:** 1 Center for Applied Genetics and Genomic Medicine, University of Arizona, Tucson, AZ 85721 USA; 2 Arizona Research Labs Division of Biotechnology, University of Arizona, Tucson, AZ 85721 USA; 3 Division of Neurosurgery, Department of Surgery, University of Arizona College of Medicine, Tucson, AZ 85724 USA; 4 Department of Surgery, University of Arizona College of Medicine, Tucson, AZ 85724 USA; 5 Department of Neurology, University of Arizona College of Medicine, Tucson, AZ 85724 USA; 6 Present Address: University of North Texas Health Science Center, Fort Worth, TX 76107 USA

## Abstract

Among patients with intractable epilepsy, the most commonly performed surgical procedure is craniotomy for amygdalohippocampectomy (AH). Stereotactic laser amygdalohippocampotomy (SLAH) has also been recently employed as a minimally invasive treatment for intractable temporal lobe epilepsy (TLE). Among patients treated with AH and SLAH approximately 65% and 54% of patients become seizure-free, respectively. Therefore, selection criteria for surgical candidates with improved prognostic value for post-operative seizure-free outcome are greatly needed. In this study, we perform RNA sequencing (RNA-Seq) on whole blood leukocyte samples taken from 16 patients with intractable TLE prior to SLAH to test the hypothesis that pre-operative leukocyte RNA expression profiles are prognostic for post-operative seizure outcome. Multidimensional scaling analysis of the RNA expression data indicated separate clustering of patients with seizure free (SF) and non-seizure-free (NSF) outcomes. Differential expression (DE) analysis performed on SF *versus* NSF groups revealed 24 significantly differentially expressed genes (≥2.0-fold change, p-value < 0.05, FDR <0.05). Network and pathway analyses identified differential activation of pathways involved in lipid metabolism, morphology of oligodendrocytes, inflammatory response, and development of astrocytes. These results suggest that pre-operative leukocyte expression profiles have prognostic value for seizure outcome following SLAH.

## Introduction

The prevalence of epilepsy in the United States is approximately 1%^1^. Among patients with epilepsy, approximately 30% are defined as medically intractable and are potential candidates for epilepsy surgery^2^. The most commonly performed surgical procedure for intractable epilepsy is craniotomy for amygdalohippocampectomy (AH) with or without anterior temporal lobectomy^3,4^. Recently, stereotactic laser amygdalohippocampotomy (SLAH) has been developed as a minimally invasive procedure for treatment of intractable temporal lobe epilepsy (TLE)^5^. Post-operative seizure freedom rates in AH and SLAH are ~65% and ~54%, respectively, with seizures persisting in the remaining patients^4,5^. Therefore, selection criteria for surgical candidates with improved prognostic value for post-operative seizure-free outcome would be of great value^6^. Traditional selection criteria for epilepsy surgery candidates have included seizure focus localization using non-invasive and invasive long-term ictal EEG recording, MRI brain and positron emission tomography brain scanning, and neuropsychological testing^7^. Yet another strategy for the selection of patients for surgery involves the identification of biomarkers to predict surgical outcome. Here we pursue an approach known as “neurosurgical genomics”, in which we employ RNA-Seq to identify gene expression profiles in patients with different seizure outcome after SLAH^8,9^.

Previous genetic profiling studies on resected tissue utilizing microarray technology identified temporal cortical and hippocampal RNA expression patterns that differed between patients with SF and NSF outcome following anterior temporal lobectomy with AH^8,9^. Given the bidirectional cellular and molecular interactions between leukocytes and the epileptic brain, systemic (peripheral) leukocytes may offer a relatively noninvasive means to assess differences in gene expression that are induced under conditions recapitulating or reflecting TLE pathophysiology. Many of these interactions involve immunosurveillance trafficking by leukocytes within epileptic brain tissue and the delivery of brain macromolecules, solutes, and immune cells to cervical lymph nodes where T-cell modulation occurs. These multimodal leukocyte/brain communications offer an opportunity to detect altered leukocyte expression profiles by virtue of the fact that leukocytes are able to access cortical tissue and flow back out to the peripheral blood. On this basis, we hypothesize that pre-operative peripheral leukocyte RNA expression parameters have prognostic value for post-operative seizure-free outcome. Ultimately, the identification of a panel of such biomarkers could be used to improve patient selection for neurosurgical operative intervention. Our differential expression (DE) data were obtained through the use of RNA-Seq, a next generation sequencing (NGS) method with improved sensitivity and dynamic range compared with microarray-based profiling^10^. Based on our results, we reiterate the concept of “neurosurgical genomics” whereby systemic leukocyte gene expression serves as a prognostic biomarker for successful outcome from operative neurosurgical intervention^11^.

## Results

### Clinical Demographics

A total of 16 consecutive patients (mean age: 39.4 years, range: 16–62 years; 10 males, 6 females) underwent comprehensive evaluation for epilepsy surgery candidacy. In this series, seven patients were rendered seizure-free and nine patients were not seizure-free following SLAH (Table 1). The temporal lobe seizure focus was localized to the left hemisphere in 9 patients, and the right hemisphere in 7 patients. Median pre-operative, baseline seizure frequency was 2 seizures per month (range: 0.25 to 60 seizures per month), with a mean of 1.4 ± 0.6 and 8.7 ± 6.4 (mean ± SEM) in the SF and NSF groups, respectively (t-test, two-tailed P value = 0.341). Seizure duration before surgery averaged 19.1 ± 6.0 years and 30.3 ± 5.8 years for the SF and NSF groups, respectively (t-test, two-tailed P value = 0.206). Post-operative SLAH seizure outcome was assessed at a mean follow-up of 18 months (range: 12 to 32 months), with a mean of 16.9 ± 2.6 and 18.3 ± 2.3 in the SF and NSF groups, respectively (t-test, two-tailed P value = 0.678). There was no significant difference in pre-operative anticonvulsant medication use between patients in the post-operative seizure-free compared to the non-seizure-free groups (Table 2). Demographic and seizure focus localization data for all patients undergoing SLAH demonstrated no significant differences for patient gender, age, baseline seizure frequency, ethnicity, MRI brain medial temporal sclerosis status, ictal surface or subdural/depth electrode EEG seizure focus localization concordance, PET scan concordance, and neuropsychological testing results between the seizure-free and non-seizure-free groups (Table 3).

**Table 1:** Patient Clinical Demographics for Stereotactic Laser Amygdalohippocampotomy (SLAH) Series.

Sample#	Gender	Age (yrs)	BSF (sz/mo)	Etiology	Outcome	Duration (yrs)	Follow-up (mos)	Laterality
1	M	38	3	Unk	SF	17	16	R
2	M	37	0.25	Unk	SF	35	15	L
3	M	60	0.25	Unk	SF	47	13	L
4	M	26	1	Unk	SF	4	13	R
5	F	32	0.33	CVA	SF	8	32	R
6	M	16	4	Unk	SF	10	12	R
7	F	35	1	Unk	SF	13	17	R
8	F	54	1	Pre	NSF	36	13	L
9	F	45	4	Abor	NSF	8	14	R
10	M	46	2	Unk	NSF	43	32	L
11	M	19	60	TBI	NSF	7	20	L
12	F	62	2	Inf	NSF	61	12	L
13	M	32	2	Unk	NSF	25	14	L
14	M	26	1	Unk	NSF	19	22	L
15	M	45	4	Unk	NSF	37	25	L
16	F	58	2	Unk	NSF	37	13	R

Etiology = Etiology of epilepsy; TBI = traumatic brain injury; Unk = unknown; CVA = stroke; Abor = abortion; Inf = infection; Pre = preeclampsia; Duration = duration of epilepsy prior to SLAH; SF = seizure-free, NSF = not seizure-free; Laterality = laterality of SLAH, L = left, R = right; SLAH = Selective Laser Amygdalohippocampotomy. BSF = baseline seizure frequency.

**Table 2:** Pre-operative Antiepileptic Medication Use of Patients for SLAH Series.

Medication	Use (Yes/No)	Seizure-Free Post-op	Not-Seizure-Free Post-op	*p* value^#^
Carbamazepine	Yes	4 (25.0%)	6 (37.5%)	1.00
	No	3 (18.8%)	3 (18.8%)	
Phenytoin	Yes	4 (25.0%)	5 (31.3%)	1.00
	No	3 (18.8%)	4 (25.0%)	
Valproic acid	Yes	1 (6.2%)	5 (31.3%)	0.145
	No	6 (37.5%)	4 (25.0%)	
Oxcarbazepine	Yes	2 (12.5%)	2 (12.5%)	1.00
	No	5 (31.3%)	7 (43.8%)	
Gabapentin	Yes	0 (0.0%)	2 (12.5%)	0.475
	No	7 (43.8%)	7 (43.8%)	
Topiramate	Yes	4 (25.0%)	1 (6.2%)	0.106
	No	3 (18.8%)	8 (50.0%)	
Phenobarbital	Yes	1 (6.2%)	4 (25.0%)	0.308
	No	6 (37.5%)	5 (31.3%)	
Zonisamide	Yes	1 (6.2%)	2 (12.5%)	1.00
	No	6 (37.5%)	7 (43.8%)	
Levetiracetam	Yes	5 (31.3%)	5 (31.3%)	0.633
	No	2 (12.5%)	4 (25.0%)	
Vigabatrin	Yes	0 (0.0%)	1 (6.2%)	1.00
	No	7 (43.8%)	8 (50.0%)	
Lacosamide	Yes	1 (6.2%)	0 (0.0%)	0.438
	No	6 (37.5%)	9 (56.2%)	
Lamotrigine	Yes	5 (31.3%)	4 (25.0%)	0.358
	No	2 (12.5%)	5 (31.3%)	
Other*	Yes	6 (37.5%)	7 (43.8%)	1.00
	No	1 (6.2%)	2 (12.5%)	

*Other includes lorazepam, zomig, clonazpam, clobazam, primidone, fycoma, temazepam, mysoline, diazepam; ^#^Fisher exact test.

**Table 3:** Demographic and Seizure Focus Localization Data of Patients for SLAH Series.

		Seizure-free	Not-Seizure-	p-value*
		Post-op	free Post-op	
Gender	Male	5	5	0.633
	Female	2	4	
Age	Mean (SD) in years	34.9	43.0	0.289^#^
		(13.3)	(14.6)	
Seizure Frequency	>2	2	3	1.000
(/month; median = 2)	≤2	5	6	
Ethnicity	Caucasian	3	4	1.000
	Hispanic/Other	2	5	
MRI Results	MTS	4	7	1.000
	Normal/other	2	3	
PET Scan	Hypometabolism /Concordant	6	6	0.229
	Disconcordant/Nonlocalizing	0	3	
Ictal Scalp EEG	Temporal Lobe/Concordant	5	10	0.375
	Nonlocalizing/Disconcordant	1	0	
Ictal Subdural/Depth EEG	Temporal Lobe Concordant	0	5	1.000
	Nonlocalizing/Disconcordant	0	0	
Neuropsychological	Lateralizing Concordant	2	4	0.567
Testing	Nonlateralizing/Disconcordant	4	2	

*Fisher Exact Test except where noted; ^#^Mann-Whitney U Test, Z = 1.06. Seizure Frequency = Baseline pre-operative seizure frequency.

Concordant/disconcordant = Concordance or disconcordance with temporal lobe treated with SLAH. MTS = medial temporal sclerosis.

### MDS Plot and Heatmap Analysis

**Figure 1:**
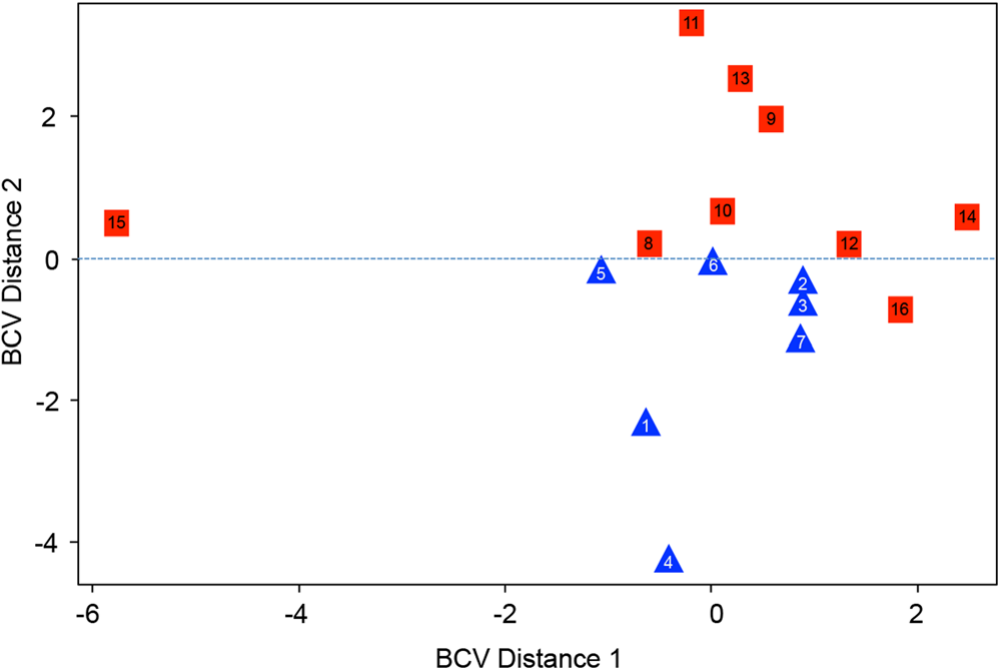
Multidimensional scaling plot (MDS) generated using edgeR showing segregation of non-seizure free patients *vs* seizure free patients PBMC transcriptional profile following SLAH. All annotated transcripts for all samples were used to generate plot. Numbered sample IDs indicate patient from list in Table 1. BCV = Biological Coefficient of Variance.

**Figure 2:**
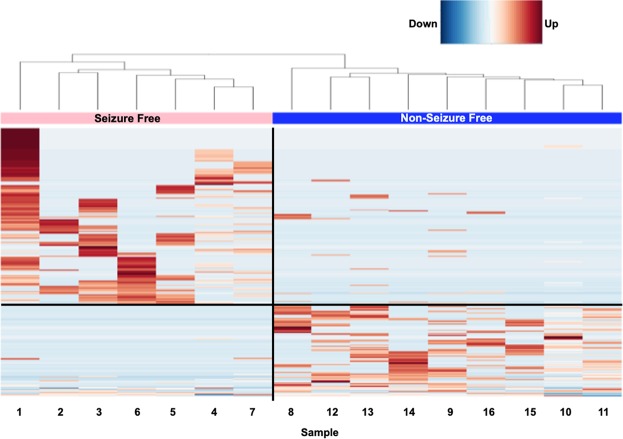
Heatmap generated in edgeR using the 250 most variable genes across samples. Unsupervised clustering showing the grouping of non-seizure free and seizure free patients. X-axis indicates sample ID from subject list in Table 1. Red indicates a higher level of comparative expression while Blue indicates a lower level of expression.

A multidimensional scaling plot (MDS) was generated using all annotated transcripts from each sample to look for segregation of outcome groups (Fig. 1). While one sample in the non-seizure free group was found to have a large biological coefficient of variation (BCV) distance from the remaining cluster of patients (sample #15), there was a general clustering and segregation of non-seizure free *vs* seizure-free outcome. Considering the variable nature of peripheral blood mononuclear cell (PBMC) expression patterns this result provided initial evidence of an outcome-predictive transcriptional profile. In an effort to reduce the general noise associated with PBMC expression, the top 250 most variable genes across samples were selected to generate a heatmap using the R package gplots (Fig. 2). This unsupervised analysis again showed a clustering of NSF *vs* SF patients with large sets of genes showing a pattern of increased expression in one patient group but not the other and vice versa (Fig. 2).

### Differential Expression Analysis

**Figure 3:**
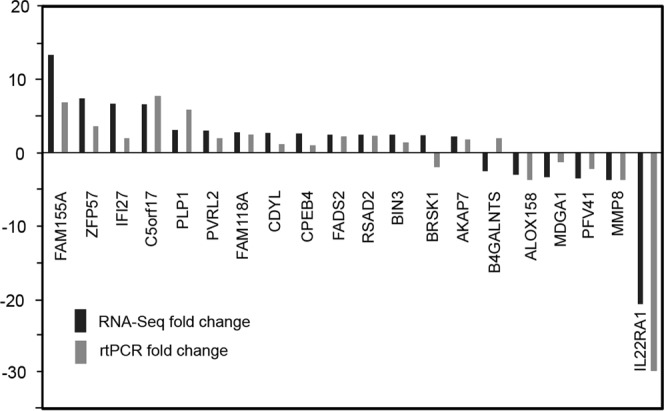
RNA-seq *vs* rtPCR fold expression validation run on all samples for all genes meeting the 2.0 FC, *p*-value < 0.05, and FDR <0.05 cutoff criteria. rtPCR assays failed to generate product for *ABCA4*, *GFAP*, *HBG1*, *BGN*.

DE analysis was performed comparing the NSF (n = 9) and SF (n = 7) patient cohorts. Based on a cutoff of 2.0-fold change (*p-*value < 0.05, FDR <0.05), 24 differentially expressed genes (DEGs) were identified. Of these, 16 transcripts were more abundant in the SF cohort while 8 were less abundant when compared with the NSF cohort (Table 4). Real time PCR (rtPCR) confirmation was performed on all samples for all genes meeting the differential expression cutoff criteria described above (Fig. 3). rtPCR assays for *ABCA4*, *GFAP*, *HBG1* and *BGN* failed to amplify product while *BRSK1* and *B4GALNT3* generated results contradictory from the RNA-seq values. The remaining 18 genes all showed confirmatory rtPCR results validating the RNA-seq data in all samples.

**Table 4:** Pre-operative Leukocyte expression NSF vs SF Outcome Following SLAH, Fold Change >2.0.

Up-Regulated	Fold-Change	FDR	p-Value	Down-Regulated	Fold-Change	FDR	p-Value
*FAM155A*	13.4	1.3E-02	9.28E-06	*IL22RA1*	−20.7	2.0E-04	4.9E-08
*ABCA4*	9.1	1.3E-02	1.00E-05	*BGN*	−15.5	5.0E-03	2.3E-06
*ZFP57*	7.4	5.8E-08	5.12E-12	*MMP8*	−4.6	1.0E-02	7.0E-06
*IFI27*	6.7	3.3E-17	1.44E-21	*PF4V1*	−3.5	3.6E-02	3.4E-05
*C5orf17*	6.7	4.5E-02	4.61E-05	*MDGA1*	−3.4	1.0E-04	1.8E-08
*PLP1*	3.2	5.0E-03	5.77E-06	*ALOX15B*	−3.1	9.0E-03	5.5E-06
*PVRL2*	3.0	1.0E-03	9.03E-08	*HBG1*	−2.7	2.0E-03	1.5E-06
*FAM118A*	2.9	5.4E-05	1.44E-08	*B4GALNT3*	−2.5	4.5E-02	6.4E-05
*GFAP*	2.8	8.0E-03	8.66E-06				
*CDYL*	2.7	2.0E-04	1.23E-07				
*CPEB4*	2.6	4.0E-04	2.16E-07				
*FADS2*	2.6	2.0E-03	1.63E-06				
*RSAD2*	2.5	1.0E-03	8.45E-07				
*BIN3*	2.5	1.0E-03	7.21E-07				
*BRSK1*	2.4	1.2E-03	1.44E-05				
*AKAP7*	1.8	3.4E-02	4.64E-05				

SF = Seizure free, NSF = Non-Seizure free, FDR = False discovery rate, SLAH = stereotactic laser amygdalohippocampotomy. Fold-change expression relative to prognostic value for seizure-free outcome following SLAH.

### Pathway Analysis

The differentially expressed leukocyte genes were first compared with reference gene lists to identify significantly overrepresented molecular functions, biological processes and pathways^12^. This test produced one marginally statistically significant result, identifying a biological process that contained 4 of the 24 differentially expressed genes in our data set (Panther overrepresentation test, p = 0.048). The four genes, *HBG1*, *FADS2*, *PLP1* and *ALOX15B* all play a role in long-chain fatty acid metabolism. We also used Ingenuity® Pathway Analysis (IPA®) to identify significantly associated biological pathways associated with seizure-free outcome. This analysis identified the following biological pathways in which clustering of these genes was significantly overexpressed: Cell morphology, lipid metabolism/molecular transport, inflammatory response/organismal injury and abnormality, and nervous system development/cellular development (Table 5).

**Table 5:** Biological Pathways Associated with Seizure Outcome Following SLAH (p < 0.001)*.

Categories	Genes
Cell Morphology	ABCA4, BGN, BIN3, BRSK1, CPEB4, FADS2, BRSK1, CPEB4, FADS2, GFAP, NECTIN2, PLP1, MMP8, ZFP57
Lipid Metabolism, Molecular Transport	ABCA4, FADS2, PLP1
Inflammatory Response, Organismal Injury & Abnormalities	ABCA4, BGN, BIN3, FADS2, GFAP, IFI27, MMP8, PLP1
Nervous System Development & Cellular Development	GFAP, PLP1

SLAH = stereotactic laser amygdalohippocampotomy. *Ingenuity® Pathway Analysis (IPA®), Qiagen.

## Discussion

**Figure 4:**
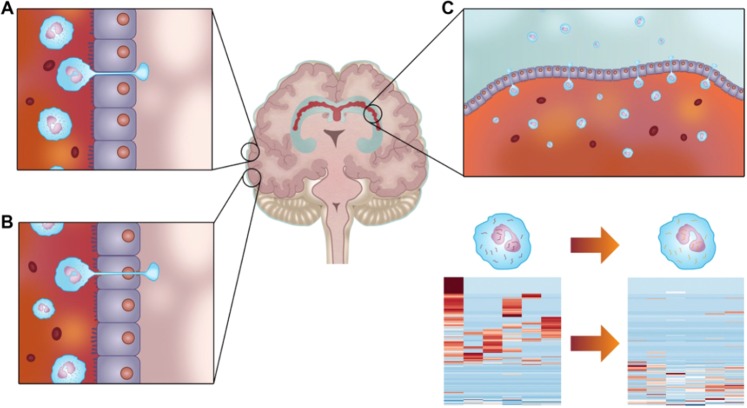
Multiple mechanisms of leukocyte trafficking in the brain. Once in the brain the leukocyte RNA expression profile is altered by the cortical environment. This altered profile is maintained once the leukocyte returns to the peripheral blood. (**A**) Human epileptic brain tissue possesses abnormal endothelial cell tight junctions with breach of the blood-brain barrier (BBB) permitting cerebral vascular endothelium intercellular leukocyte entry into the brain. (**B**) With epilepsy-associated neuroinflammation, leukocyte trafficking across the BBB also occurs via cerebral vascular endothelial transcellular diapedesis. (**C**) Choroid plexus vascular endothelium intercellular leukocyte entry into the cerebral spinal fluid. Leukocyte RNA transcription profile change from exposure to cerebral molecular microenvironment producing genomic transformation recapitulating temporal lobe epilepsy pathophysiology in peripheral blood leukocyte (bottom right).

In this study we employed RNA-Seq to identify preoperative peripheral leukocyte gene expression profiles in TLE patients undergoing SLAH in order to test the hypothesis that these RNA expression profiles are prognostic for post-operative seizure-free outcome. We found that peripheral leukocyte RNA expression patterns differentiated patients with SF and NSF outcomes, with 24 transcripts differing by ≥2-fold between these patient groups. To fully explore the question of whether circulating leukocytes may express different gene expression profiles as a result of their presence in the blood streams of TLE patients with different proclivities for SF or NSF surgical outcomes, it is important to first examine the varied means by which immune cells are recruited and infiltrated into the epileptic brain (Fig. 4). Historically, the brain was considered an immunologically-privileged organ^13^. However, it is now known that the brain is immunologically active^14,15^. Experimental and clinical evidence support the concept of a link between epilepsy and systemic and central nervous system inflammation, both of which impact seizure susceptibility^16,17^. Inflammation is significantly involved in the pathophysiology of epilepsy and inflammatory mediators are produced by neurons, astrocytes, and microglia^18^. With activated leukocytes being shown to infiltrate the brain in several forms of human epilepsy, as well as evidence for significant blood-brain barrier (BBB) disruption, there is opportunity for leukocyte communication with brain cells^19,20^. This communication between leukocytes and neuronal tissue can influence both epileptogenicity and seizure onset^20,21^. Human epileptic brain tissue also demonstrates abnormal endothelial cell tight junctions with breach of the BBB, thus allowing WBC infiltration of the perivascular spaces of the disrupted BBB^14,15^ (Fig. 4). In addition, during neuroinflammation, leukocyte trafficking across the BBB can also occur via transcellular diapedesis^22^ (Fig. 4). Seizure-produced BBB disruption also increases natural killer (NK) cell migration into brain tissue, which further contributes to cerebral inflammation in epileptic foci^16^. This immunopathogenesis of epilepsy involves reciprocal endothelial-leukocyte interactions in the context of BBB disruption^15^. Furthermore, the endothelial-immune cell bidirectional interactions that modulate leukocyte migration into the brain are coordinated by immunoglobulin endothelial cell adhesion molecules and leukocyte integrins^15^. All of the above-mentioned mechanisms of immune cell migration into the brain provide an immunosurveillance function under both normal conditions and during neuroinflammatory pathologies such as TLE^15^.

Of the 24 differentially expressed genes in the leukocytes of TLE patients with SF *versus* NSF outcomes, four are associated with long-chain fatty acid metabolism: *ALOX15B* (FC: −3.1), *FADS2* (FC: +2.6), *PLP1* (FC: +3.2), and *HGB1* (FC: −2.7). *ALOX15B* (arachidonate 15-lipoxygenase, type B) is an interesting candidate biomarker that is down-regulated in association with post-SLAH seizure-free outcome in leukocytes (Table 4). *ALOX15B* encodes a 15-lipoxygenase that oxidizes fatty acids to inflammation-promoting substances^23^ and is responsible for regulating IL-12 mediated chronic inflammation and inflammatory activity through IL-1β and TNF-α^24,25^. Biological membranes, which consist of phospholipids containing polyunsaturated fatty acids, are very susceptible to ROS oxidation, known as lipid peroxidation^26^. There is also evidence for an association between mitochondrial oxidative stress and dysfunction both as a result of seizures and as a contributor to epileptogenesis^27^. Down-regulation of *ALOX15B* may contribute to post-SLAH seizure freedom through an anti-inflammatory reduction of fatty acid oxidation, thus inhibiting epileptogenicity.

Further supporting this concept, it is widely known that metabolism-based therapy for medically-refractory epilepsy has included generation of fatty acid oxidation products known as ketone bodies. These ketone bodies serve as alternatives to glucose fuel for the brain in the high-fat, low carbohydrate and low-protein antiseizure ketogenic diet. The ketogenic diet has been shown to decrease mitochondrial ROS, increase cellular antioxidant capacity, and prevent mtDNA deletions and cell death^28^. Ketone bodies possess neuroprotective activity, decrease reactive oxygen species (ROS), and increase cellular adenosine triphosphate (ATP) levels^28^. Evidence from a rodent model of TLE links ketone body suppression of epilepsy and cognition enhancement to the mitochondrial permeability transition (mPT) which regulates ROS and ATP levels and cell death pathways^28^. The ketogenic diet also generates increased plasma decanoic acid, which produces anti-seizure activity through inhibition of excitatory neurotransmission in *ex vivo* rat hippocampal slice models^29^. The anti-seizure effect of decanoic acid occurs through direct inhibition of the AMPA (α-amino-3-hydroxy-5-methyl-4- isoxazolepropionic acid) glutamate receptor whose most abundant subunits are GluA1 and GluA2^29,30^. Astrocytes have important roles in the reuptake and recycling of synaptic glutamate during conditions of systemic inflammation, including involving changes in hippocampal synaptic function^30^. GFAP is an important intermediate filament protein essential for structural integrity of astrocytes and may be compromised during conditions of systemic inflammation and blood-brain barrier disruption, such as occur in TLE^30^. For instance, hippocampal detection of the pro-inflammatory cytokine, IL-6, whose cell surface receptor is expressed on astrocytes, is followed acutely by decreased mRNA GFAP expression^30^. In a rodent model of increased hippocampal and systemic IL-6, GluA1 and GluA2 demonstrated significant hippocampal post-synaptic increase and decrease, respectively^30^. Changes in cellular calcium entry are regulated by the GluA1 subunit, which is highly permeable to calcium, while decreased GluA2 may increase the likelihood of glutamate release^30^. From a mechanistic standpoint, the finding in the current study that increased leukocyte GFAP RNA expression is prognostic for post-SLAH seizure freedom is consistent with an anti-seizure effect of GFAP associated with reduced AMPA-GluA2 mediated glutamate release, reduced extracellular glutamate and decreased glutamate-related excitotoxicity^31^.

Additional confirmation of the role of lipid metabolism as a biomarker comes from the IPA analysis based on the same 24 significantly differentially expressed genes in the patient PBMCs associated with post-SLAH seizure-free outcome (Table 5), which also identified cell morphology, inflammation, nervous system, and cell development as key biological pathways associated with seizure outcome. The identification of these biological processes is particularly interesting as epileptogenesis involves activation of the central nervous and systemic immune systems, which in turn can be modulated by lipid metabolism pathways^32^. As mentioned above, *PLP1* and *FADS2* transcripts were more abundant in association with post-SLAH seizure-free outcome while IPA identified a third gene involved in lipid metabolism and transport that was also more abundant in the SF cohort (*ABCA4* FC: + 9.1). *ABCA4* uses ATP to transport a variety of different substrates including the phospholipids, phosphatidylcholine and phosphatidylethanolamine, across biological membranes^33,34^. Both phosphatidylcholine and phosphatidylethanolamine are significantly depleted by seizure activity^35^. ATP-binding cassette transporters are critical to the integrity of the central nervous system as key regulators of cellular lipid transport processes, thus maintaining membrane lipid symmetry^36,37^. Conversely, the loss of *ABCA4* activity results in accumulation of lipid debris and defective phagosome processing^38^. The importance of maintaining potentially excitable membrane lipid symmetry is again underscored by the anti-epileptic effects of fatty acid nutritional supplementation. Although more studies are needed, clinical trial evidence currently suggests that supplementation with omega-3 fatty acids may decrease the frequency and duration of seizures and enhance quality of life in patients with epilepsy^32,39,40^. Given this evidence, the role of increased *ABCA4* expression may likely prove protective against seizure activity.

Likewise, there is functional evidence suggesting that both *PLP1* and *FADS2* may play a role in the outcome following SLAH. Synaptic transmission and neuronal excitability are regulated by myelination, and hippocampal demyelination has been detected in TLE^41^. *PLP1* expression is known to counteract this loss of myelination and is able to prevent oligodendroglial cell loss^42,43^. Additionally, *FADS2* is known to mediate direct fatty acid desaturation to yield docosahexaenoic acid (DHA) in human cells^44^. DHA is an omega-3 fatty acid that is highly abundant in neuronal membranes^45^. DHA is involved in synaptic membrane function and modulates glutamate availability by inhibiting its transporter, glutamate/aspartate transporter (GLAST)^46^. DHA also regulates GABA receptor subunits; raises the seizure threshold in mice and rats; prolongs seizure latency; reduces the amount of rescue-medication required during antiseizure therapy; reduces epileptic activity through frequency-dependent blockade of sodium channels; and has been shown *in vitro* to decrease hippocampal excitability through CA3 circuitry^45–47^. DHA anti-epileptic activity may also be involved though modulation of neurotransmitter receptors, ion channels and regulation of synaptic plasticity while also decreasing neuronal excitability and potentiating GABAergic activity^45,46^. Further evidence has been shown through systemically administered DHA which was able to inhibit kindling progression and electrically induced hippocampal hyperexcitability associated with evoked seizures, thus limiting progression of limbic seizures in a rodent model of TLE^46^. Higher levels of functional DHA mediated by *FADS2* expression would again argue for a mechanistically protective effect for prospective patients. Additionally, the derivative of DHA resulting from seizures and phospholipase A2 activity, neuroprotectin 1 (NPD1), inhibits hippocampal evoked epileptiform activity and motor seizures^46^. It is also anti-inflammatory through down-regulation of proinflammatory cytokines and is protective against oxidative stress^46^.

Taken together these results suggest that over-expression of leukocyte genes in biological pathways supporting lipid metabolism, function, and transport, as well as inhibition of genes regulating fatty acid oxidation, are prognostic for post-SLAH seizure-free outcome. As lipid metabolism and transport are critical to normal central nervous system health and function, these findings of significant pathway associations fit with a mechanistic view of a seizure-free outcome. Specifically, up-regulation of *ABCA4* and *PLP1* may promote post-SLAH seizure freedom through maintenance of central nervous system membrane lipid symmetry and myelinated oligodendroglial cell populations, respectively. Down-regulation of *ALOX15B* and up-regulation of *FADS2* may further support post-SLAH seizure-free outcome through reduction of inflammatory fatty acid oxidation and enhancement of DHA anti-epileptic activity, respectively.

This study was intended to serve as an initial proof of concept to generate further data and hypotheses around the concept of neurosurgical genomics. As such, there were numerous limitations, not the least of which was that while clinical data were acquired prospectively to evaluate the neurosurgical genomic hypothesis all research was performed at a single institution limiting the generalizability of the results. While the leukocyte RNA expression data were sufficient in this small sample to produce univariate gene expression parameters predictive of post-SLAH seizure freedom, the sample size was not large enough and the data were not sufficient to establish a multivariate model. A larger prospective, multi-center clinical study will be required to confirm prognostic leukocyte RNA expression parameters predictive of post-SLAH seizure-free outcome.

In conclusion, next-generation sequencing and differential expression analyses performed in 16 consecutive patients with intractable TLE demonstrate leukocyte-RNA expression patterns predictive of seizure-free outcome following SLAH. The results suggest that it may be possible to develop a profile of leukocyte gene expression prognostic for seizure-free outcome following SLAH. This could have the effect of improving the selection of candidates for SLAH, and support further development of the concept of “neurosurgical genomics” by which pre-operative leukocyte gene expression may predict the desired response to neurosurgical operative intervention.

## Methods

### Patient Population

This study involves a consecutive series of 16 patients evaluated at the Arizona Comprehensive Epilepsy Program at Banner University Medical Center - Tucson for intractable TLE. All patients met the Task Force of the ILAE (International League Against Epilepsy) Commission on Therapeutic Strategies definition of drug-resistant epilepsy. Therefore, each patient had “intractable epilepsy” resistant to at least two well tolerated, appropriately chosen and prescribed anti-epileptic drug regimens either as mono-therapies or in combination^11,48^. This study was approved by and conducted in accordance with the approved protocols and subject consent forms provided by the University of Arizona College of Medicine Institutional Review Board. Informed consent was obtained from all participants in the study.

### Seizure Focus Localization

**Figure 5:**
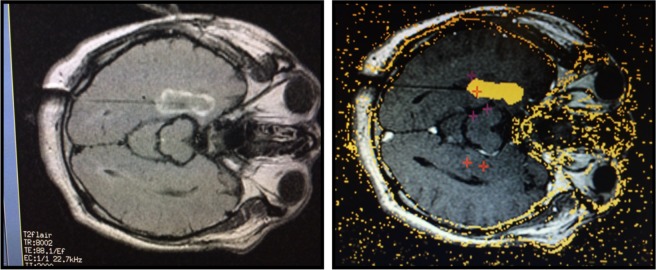
Real time T2-FLAIR (left) and thermal (right) MRI brain images of immediate post-operative brain demonstrating blood brain barrier disruption outline and of permanent brain tissue destruction during stereotactic laser amygdalohippocampotomy (SLAH) (orange).

All 16 patients underwent Phase I and, where appropriate, Phase II evaluation for epilepsy surgery candidacy as previously described^4^ (Fig. 5). Phase I evaluation may include long-term surface ictal EEG recording, MRI brain scanning, PET brain scanning and neuropsychological testing. For patients in whom Phase I long-term scalp-EEG recording failed to localize the ictal seizure focus, Phase II evaluation included long-term subdural and/or depth EEG recording. In all patients, the ictal seizure focus was localized to a single temporal lobe.

### Seizure Focus Ablation and Seizure Outcome

In all 16 patients, based on seizure focus localization, SLAH was performed as previously described^49^. The neurosurgical operative technique for SLAH involves stereotactic planning of an occipital to amygdalohippocampal trajectory for MRI-thermal guided laser ablation (Visualase™, Medtronic, Dublin, Republic of Ireland) AH employing between 3 to 5 thermal ablation isocenters per patient. The goal is optimal thermal ablation of the amygdala and hippocampus, from the amygdala anteriorly to the hippocampus at least at the level of the tectum posteriorly. All patients were evaluated at a minimum of 12-months follow-up on anticonvulsant medication to determine post-operative seizure outcome defined as “seizure-free” or “not seizure-free” (Table 1). Patients experiencing “auras only” were classified as “not seizure-free”. A patient having a rare seizure due to anticonvulsant medication non-compliance followed by prolonged resumption of seizure freedom associated with anticonvulsant medication compliance was classified as “seizure-free”.

### Leukocyte RNA Acquisition and Expression

Pre-operative whole blood was obtained immediately prior to placement of the stereotactic head frame before SLAH and was stored in RNA stabilization Solution (Qiagen, Valencia, CA) at −80° C until RNA extraction was performed. Total RNA was extracted from leukocytes using RNeasy lipid tissue mini kit (Qiagen, Valencia, CA) following manufacturer’s instructions. First-strand cDNA was prepared with the SuperScript III kit (Life Technologies/Thermo Fisher Scientific, Carlsbad, CA). RNA Samples were assessed for quality with a High Sensitivity RNA Analysis Kit (Fragment Analyzer; Advanced Analytical Technologies, Ankeny, IA). Concentration was determined using a Quant-iT RiboGreen RNA Assay Kit (Molecular Probes; Thermo Fisher Scientific, Carlsbad, CA). RNASequence (RNA-Seq) Libraries were constructed using a stranded mRNA-Seq Kit (TDS KR0960 – v3.15; KapaBiosystems, Wilmington, MA). After completion, quality and average fragment size were assessed with the Fragment Analyzer (Advanced Analytical Technologies, Ankeny, IA). Concentration was determined with the Illumina Universal Adaptor-specific qPCR kit (KapaBiosystems, Wilmington, MA). Equimolar samples were pooled and clustered for sequencing on the HiSeq. 2500 (Illumina, San Diego, CA). Sequencing was performed using Rapid-Run SBS 2 × 100bp chemistry (Illumina, San Diego, CA) as previously described^50^.

### Sequence analysis

Sample data were demultiplexed, trimmed and quality filtered using Trimmomatic (USADelLab, Aachen, Germany). Fastq files were splice aligned against the GRCh37 reference genome using STAR aligner version 2.5.2b^51^. Gene expression counts were obtained using htseq-count version 0.6.1^52^. Both splice alignment and counting were performed with Ensembl Annotation of the NCBI reference genome and raw counts analyzed with edgeR version 3.16.5^53^.

### Differential expression analysis

Differential expression was analyzed in edgeR, version 3.16.5, in R edgeR’s exactTest function. Gene expression counts were first normalized using the calcNormFactors function, which uses the trimmed mean of M values (TMM) to create a set of scaling factors that eliminates composition biases between sample libraries. Due to the variance between samples, the trended dispersion (the dispersion calculated from a gene’s abundance) was used for the exactTest calculation.

### Quantitative reverse transcriptase polymerase chain reaction (qRT-PCR)

RNA from peripheral blood was isolated for each patient using the PAXgene blood RNA kit (Qiagen, Hilden, Germany) and cDNA was generated with the SuperScript III kit (Life Technologies/Thermo Fisher Scientific, Carlsbad, CA). Taqman probes were obtained from Life Technologies for the genes as validated using control cDNAs. Taqman reactions were performed in triplicate in a 15uL reaction volume using the Taqman Fast Advance Master Mix (Thermo Fisher Scientific, Carlsbad, CA). All reactions were run on an ABI 7900HT using the SDS 2.4 software (Life Technologies/Thermo Fisher Scientific, Carlsbad, CA) with ABI384 well Optical PCR plates and AB-1170 Optical PCR film (Fisher Scientific International, Inc., Hampton, NH). All samples were run with the endogenous control GAPDH probe set (Life Technologies/Thermo Fisher Scientific, Carlsbad, CA). Differential expression analysis was performed using the standard delta-delta CT method^54^.

### Pathway and enrichment analysis

Differentially expressed transcripts were analyzed for enrichment of GO terms using the Overrepresentation Test (release 13.1)^12^, accessing the GO Ontology database. Molecular process, cellular component and biologic process annotation databases were also used. Ingenuity® Pathway Analysis (IPA®) of all differentially expressed leukocyte genes predictive of seizure-free outcome was performed to further identify biological pathways (categories) involving diseases and functional annotations in which clustering of seizure outcome associated genes were significantly over expressed (Qiagen, Hilden, Germany).

### Feature selection

RNA-seq has some major advantages over microarrays such as providing less noisy data and detecting novel transcripts and isoforms. The first property can improve the predictive performance of classification algorithms, while the second may reveal biomarkers that are tissue specific or that were previously not known to exist. The objective is to find a predictive model that uses expression data from a set of genes that show significantly different expression patterns (features) to calculate a score that correlates with how likely a person with a particular profile is to have a seizure-free outcome after SLAH. Our learning approach addresses some of the challenges that come from large-scale genomic datasets. Whole transcriptome (RNA-Seq) data were analyzed for prognostic value for seizure-free outcome following SLAH. We utilized multivariate logistic regression as our model of gene expression associated with seizure-free post-surgery outcome. The receiver-operating characteristic assessed the quality of our classification. The important features formed the basis for assessing the most informative markers for blood testing. We present a prioritized subset of genes and prognostic models to aid neurophysiologists, epileptologists, and epilepsy surgeons in understanding the molecular mechanisms of TLE, to develop better potentially predictive models for selection of SLAH candidates, and to possibly improve post-surgical seizure outcome.

### Ethical Approval

All procedures performed in studies involving human participants were in accordance with the ethical standards of the University of Arizona Institutional Review Board and with the 1964 Helsinki declaration and its later amendments or comparable ethical standards. IRB# 1401194084, The University of Arizona.
